# Household food insecurity during pregnancy as a predictor of anthropometric indices failures in infants aged less than 6 months: a retrospective longitudinal study

**DOI:** 10.1017/S1368980021003591

**Published:** 2022-04

**Authors:** Karim Karbin, Fatemeh Khorramrouz, Ehsan Mosa Farkhani, Seyyed Reza Sobhani, Negin Mosalmanzadeh, Zahra Shahriari, Golnaz Ranjbar

**Affiliations:** 1 Faculty of Medicine, Mashhad University of Medical Sciences, Mashhad, Iran; 2 Department of Nutrition, Faculty of Medicine, Mashhad University of Medical Sciences, Mashhad, Iran; 3 Department of Epidemiology, School of Public Health, Mashhad University of Medical Sciences, Mashhad, Iran; 4 Department of Nutrition Sciences, Varastegan Institute for Medical Sciences, Mashhad, Iran; 5 Faculty of Medicine, Mashhad University of medical Sciences, Mashhad, Iran

**Keywords:** Food Insecurity, Pregnancy, Infants, Stunting

## Abstract

**Objective:**

To investigate the impact of household food insecurity during the third trimester of pregnancy on the growth indicators of infants aged less than 6 months.

**Design:**

Retrospective longitudinal study.

**Setting:**

137 healthcare centres (15 cities) in Khorasan Razavi province, Iran. Data were extracted from the Sina Electronic Health Record System (SinaEHR®).

**Participants:**

This study was conducted on 2481 mother and infant dyads during November 2016–March 2019. The Household Food Insecurity Access Scale (nine-item version) was used to measure food insecurity in the third trimester of pregnancy. Women who delivered singleton infants were included in the study, and anthropometric indices of infants were measured throughout the first 6 months of life.

**Results:**

Approximately 67 % of the participants were food secure, while 33 % had varying degrees of food insecurity. The children born to the mothers in the food-insecure households were, respectively, 2·01, 3·03, and 3·83 times more likely to be stunted at birth (95 % CI 1·17, 3·46), 4 months (95 % CI 1·21, 7·61) and 6 months of age (95 % CI 1·37, 10·68) compared to their counterparts in the food-secure households. However, there were no significant differences in mean birth weight, birth height and head circumference at birth between the two groups.

**Conclusions:**

Household food insecurity during pregnancy is a risk factor for stunting in infants aged less than 6 months. Therefore, national nutrition programs could considerably support women in food-insecure households during and before pregnancy.

Food security is defined as the physical, social and economic accessibility of sufficient, safe and nutritious foods all the times for people to meet their dietary needs and food preferences for active and healthy life^(1)^. In 2018, the FAO report showed that approximately two billion people across the world are disposed to moderate or severe food insecurity^(2)^. In Iran, the prevalence rate of food insecurity among households, mothers and children has been reported to be 49, 61 and 67 %, respectively, with an increasing trend during 2004–2015^(3)^.

It is believed that food insecurity mainly affects underprivileged societies, young children and women of the reproductive age^(4,5)^. Ample evidence suggests that food insecurity is associated with poor health outcomes in women, including higher risk of obesity^(6)^, higher gestational weight gain^(6,7)^, anaemia^(8)^, depression^(9)^, gestational complications (diabetes, hypertension and birth defects)^(8,10,11)^ and the ultimate increase in the burden of healthcare costs on the community^(2)^. Due to increased nutritional requirements during pregnancy, not reaching the adequate nutrient supply may increase the risk of malnutrition and consequent intrauterine growth restrictions^(12,13)^ which is a significant risk factor for under-five morbidities and mortalities^(14)^.

Extensive research has been focussed on food insecurity, and some of the findings have confirmed the impact of maternal food insecurity on infancy and early childhood growth^(15,16)^. In this regard, a study conducted in the USA indicated that maternal food insecurity was associated with a threefold increase in the delivery of low-birth weight infants^(17)^. Also, living in food-insecure households severely affects the health status of children in later life as they are more prone to malnutrition in growth age. Not only may food insecurity lead to increased odds of childhood obesity^(18)^ and poor cognitive performance^(19,20)^, but it also is associated with psychological distress during their transition into adulthood^(21)^.

Food insecurity is assumed to affect the growth of infants by feeding practices such as breastfeeding. As the most cost-effective approach to meeting the nutritional needs of infants and reducing childhood diseases^(22)^, exclusive breastfeeding in the first 6 months of life is adversely affected by food insecurity through changes in the initiation and duration of breastfeeding^(23)^; therefore, food insecurity seems to adversely impact infant growth variably^(9,24)^. Nevertheless, only limited studies have been focussed on the effect of household food insecurity (HFI) on infant growth indices, proposing conflicting results^(25)^.

This study aimed to investigate the effects of HFI during the third trimester of pregnancy on the growth indicators of infants aged less than 6 months.

## Materials and Methods

### Study design, sampling and inclusion criteria

In this retrospective longitudinal study, data were collected from Sina Electronic Health System (SinaEHR®) during November 2016–March 2019. SinaEHR is an integrated health information system supervised by Mashhad University of Medical Sciences, which contains the health records of more than five million people in Khorasan Razavi province, Iran.

The current research participants were selected from 15 cities (137 healthcare centres) in Khorasan Razavi province among women 18–45 years of age who referred to the healthcare centres for routine check-ups during their third trimester of pregnancy. To maximise the target population (registered in SinaEHR), we pooled data between November 2016 and March 2019, and random sampling was used for selecting study participants. We included only women who delivered singleton infants, but mothers with a history of chronic heart diseases, anaemia, type II diabetes mellitus and gestational diabetes were excluded. In addition, we excluded infants with a history of hospitalisation during the first 6 months of life.

### Sociodemographic and anthropometric measurements

The sociodemographic and anthropometric data, including age, education level, smoking during pregnancy, medical history of the mother and infant, place of residence and maternal pre-pregnancy BMI were collected using a questionnaire which was developed and applied via the SinaEHR. Anthropometric variables of infants (weight, height, and head circumference at birth, second, fourth and sixth months of life) and their mothers (weight and height) were measured by trained personnel using standard protocol.

Infants’ weight was measured with minimal clothing using a Seca scale with an accuracy of 10 g (Seca 725, Hamburg, Germany). Their height (supine position) and head circumference were also measured using a standard measuring tape with 1 mm’s accuracy. Maternal weight and height were measured with light clothing and no shoes using a Seca scale and a stadiometer (Seca 755, Hamburg, Germany) with an accuracy of 1 mm for height and 100 g for weight.

The infants who weighed less than 2500 g were considered low birthweight, and those weighing ≥4000 g were classified as large for gestational age^(26)^. The WHO Z score system was used to classify the nutritional status, and scores less than −2 of the WHO standards for height-for-age Z scores (HAZ), weight-for-height Z scores and weight-for-age Z scores (WAZ) were considered as stunted, wasted and underweight, respectively. Regarding the weight-for-height Z score standard, infants with the Z scores between −2 and 2 were considered normal, and those with the Z score of above 2 were considered overweight. In addition, infants with the HAZ between the Z-score lines −1 and 2, WAZ between the Z-score lines of −1 and 3 were classified as normal^(27)^.

### Food insecurity measurement

HFI was measured using a standard questionnaire (Household Food Insecurity Access Scale, Household Food Insecurity Access Scale), which has been developed by Food and Nutrition Technical Assistance II project in collaboration with others^(28,29)^. The validity of the Persian version of this questionnaire has been confirmed by Salarkia et al., who reported the acceptable internal consistency of the scale for Iranian households with the Cronbach’s alpha of 0·95^(30)^.

The Household Food Insecurity Access Scale is composed of a set of nine items specific to an experience of food insecurity occurring within the previous 4 weeks. Endorsed a standard scoring procedure was used with 1 point for occurrence and 0 for non-occurrence. The frequency scores are within the range of 0–3, with zero indicating non-occurrence, one showing rare occurrence (once/twice in the past 4 weeks), two indicating sometimes (3–10 times in the past 4 weeks) and three for often (>10 times in the past month)^(31,32)^. For the purpose of this article, based on the Household Food Insecurity Access Scale total score (nine items based on the frequency score), the study population was classified as food secure (scores 0–1), mildly food insecure (scores 2–7), moderately food insecure (scores 8–14) and severely food insecure (scores 15–27).

### Statistical analysis

Data analysis was performed in SPSS version 22. The continuous variables were expressed as mean and sd, and the categorical variables were expressed as frequency and percentage. Independent samples *t*-test was used to assess the significant difference in the Z scores between the food-secure and food-insecure subjects, and one-way repeated-measures ANOVA was applied to evaluate the significant difference in the Z scores between various measurement times. The variables were categorised, and the relative risk was calculated at 95 % CI^(12)^. In addition, multinomial logistic regression was applied considering food insecurity as a factor and the covariates of maternal age, pre-pregnancy BMI, education level and parity. In all the statistical analyses, the *P*-value of less than 0·05 was considered significant.

## Results

**Fig. 1:**
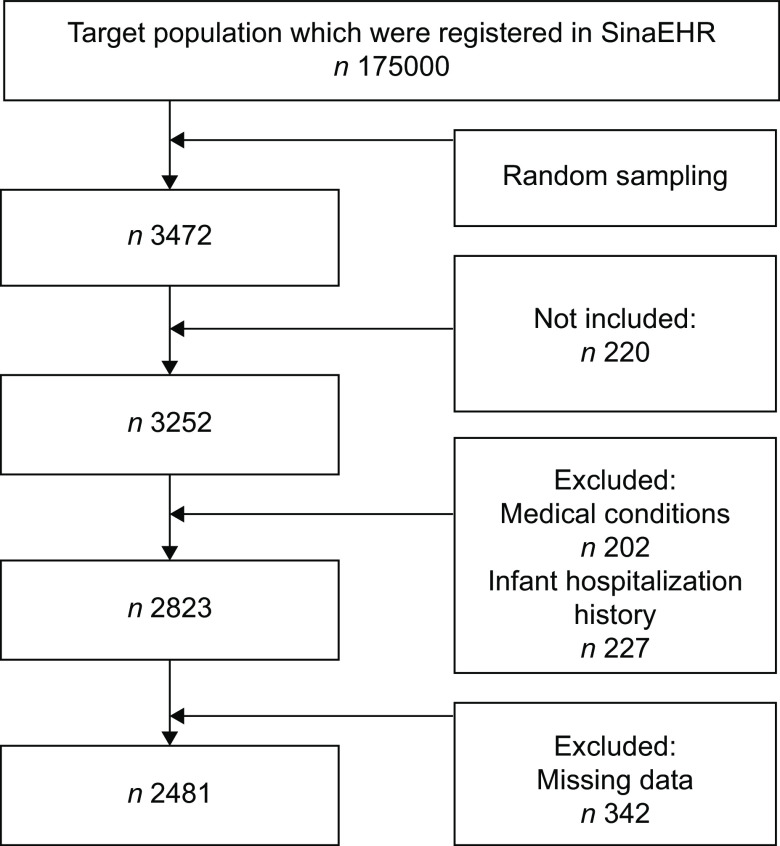
Flowchart of sampling procedure

As shown in Fig. 1, more than 175 000 pregnant women were registered in SinaEHR during the study period, and 3474 women were randomly selected for this study. Among these women, 3252 were aged 18–45 years and had live, singleton deliveries. Notably, 202 women were excluded due to medical conditions (e.g. type II diabetes mellitus, gestational diabetes mellitus, anaemia, CHD), 342 women were excluded due to data loss and 227 cases were excluded due to neonatal hospitalisation history.

The sociodemographic characteristics and anthropometric indices of the participants are presented in Table 1. The proportion of the infants by gender was reported at 50 %. In addition, 0·8 % of the women (*n* 20) had smoking habits within the past 12 months upon enrolment. The majority of study population were urban (95·6 %) and outskirts residents (49·7 %). In terms of education level, 37·2 % of the participants had an academic degree, 35·4 % had a high school diploma and the others had secondary education or were illiterate. Approximately 4 % of the neonates (*n* 100) were low birth weight, and 4·5 % (*n* 110) had macrosomia (weight: >4000 g). The infants’ mean birth weight was 3237 g, the mean pre-pregnancy BMI was 26·29 kg/m^2^ and the mean gravidity was 2·44.

**Table 1:** Sociodemographic characteristics of sample population (number of percentage and mean and sd values)

		*N* (%)		Mean±sd	
Variables		*N*	%	Mean	sd
Gender	Male	1280	51·6		
	Female	1201	48·4		
Birth weight (g)	LBW	100	4	3237	439
	Normal	2270	91·5		
	Macrosomia	111	4·5		
Birth height (cm)				49·93	2·33
Birth head circumference (cm)				34·74	1·74
Maternal age (year)				29·52	5·78
Pre-pregnancy BMI				26·29	5·40
Mode of delivery	C-section	1163	46·9		
	Natural	1318	53·1		
Premature birth		42	1·7		
Gravidity				2·44	1·39
Exclusive breastfeeding		1988	80·1		
Smoker mothers		20	0·8		
Maternal education level	Academic	923	37·2		
	Diploma	878	35·4		
	Below Diploma	680	27·4		
Place of residence	Rural	110	4·4		
	Town (<20 K)	13	0·5		
	Town (20–50 K)	164	6·6		
	Town (50–500 K)	82	3·3		
	Outskirts	1233	49·7		
	City centre	879	35·4		

As presented in Table 2, the highest prevalence of wasting was observed at birth, which gradually decreased with infants’ growth. The comparison of the mean Z scores between the food-secure and food-insecure subjects using independent *t*-test (Table 3) indicated that birthweight, birth height, head circumference at birth and birth’s Z scores were not significantly different between groups, although the infants in food-secure households had lower WAZ at 6 months of age (*P* = 0·003) and lower HAZ at the age of 2, 4 and 6 months (*P* ≤ 0·001).

**Table 2:** Prevalence of classified levels of Z scores (number of percentage (%) values)

		Birth *N* (%)		Two Months *N* (%)		Four Months *N* (%)		Six Months *N* (%)	
Characteristics		*N*	%	*N*	%	*N*	%	*N*	%
WHZ	Wasted	203	8·2	102	4·1	74	3	62	2·5
	Normal	2238	90·1	2295	92·4	2320	93·5	2320	93·5
	Overweight	40	1·6	84	3·4	87	3·5	99	4
WAZ	Underweight	67	2·7	72	2·9	44	1·8	40	1·6
	Normal	2374	95·7	2335	94·1	2355	94·9	2342	94·4
	Other	40	1·6	74	3	82	3·3	99	4
HAZ	Stunted	97	3·9	77	3·1	30	1·2	27	1·1
	Normal	2377	95·8	2384	96·1	2436	98·1	2432	98
	Other	7	0·3	20	0·8	15	0·6	22	0·9

Z scores of HAZ, WHZ and WAZ blow −2 considered stunted, wasted and underweight, respectively; WHZ of −2 to 2 considered normal and >2 classified as overweight; HAZ of −1 to 2 and WAZ of −1 to 3 considered normal. HAZ, height-for-age Z scores; WAZ, weight-for-age Z scores; WHZ, weight-for-height Z scores.

**Table 3:** Independent samples *t*-test for comparison of Z scores in food-secure and food-insecure subjects (mean and sd values)

Characteristics	Food-secure		Food-insecure		t	df	*P*-value
	Mean ± sd		Mean ± sd				
	Mean	sd	Mean	sd			
Birth weight (cm)	3247	431	3218	456	−1·5	2479	0·054
Birth height (cm)	49·96	2·25	49·88	2·49	−0·76	2453	0·057
Birth head circumference (cm)	34·75	1·68	34·72	1·86	−0·52	2436	0·177
Birth WHZ	−0·40	1·17	−0·40	1·20	0·04	1893	0·971
2-month WHZ	−0·05	1·12	0·00	1·14	1·14	2375	0·254
4-month WHZ	−0·05	1·12	0·02	1·05	1·32	2310	0·186
6-month WHZ	0·10	1·10	0·09	1·06	−0·33	1854	0·745
Birth WAZ	−0·16	0·87	−0·21	0·94	−1·22	1234	0·221
2-month WAZ	0·09	0·98	0·01	1·03	−1·83	2367	0·067
4-month WAZ	0·18	1·02	0·10	1·01	−1·79	2311	0·073
6-month WAZ	0·27	1·02	0·12	1·01	−2·92	1848	**0·003**
Birth HAZ	−0·08	1·05	−0·16	1·11	−1·56	1891	0·120
2-month HAZ	0·24	1·17	0·05	1·25	−3·59	2379	**<0·001**
4-month HAZ	0·40	1·08	0·23	1·12	−3·46	2311	**0·001**
6-month HAZ	0·47	1·09	0·23	1·10	−4·43	1853	**<0·001**

HAZ, height-for-age Z scores; WAZ, weight-for-age Z scores; WHZ, weight-for-height Z scores.

*P* < 0·05 considered statistically significant.

**Fig. 2:**
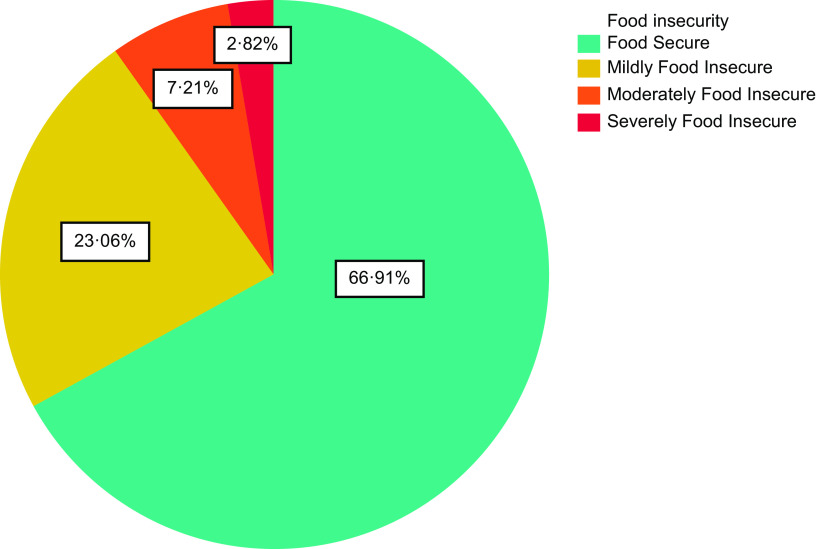
Prevalence of household food insecurity during pregnancy

Figure 2 shows the prevalence of food insecurity in the sample population. As illustrated herewith, approximately two-thirds of the participants were food secure, while the prevalence of mild, moderate and severe food insecurity was 23 % (*n* 572), 7 % (*n* 179) and 3 % (*n* 70), respectively. Table 4 shows the relative risk of HFI during pregnancy for the anthropometric characteristics of the infants. Accordingly, the children born to the women in food-insecure households were, respectively, 1·96, 2·72, and 3·87 times more likely to be stunted at birth (95 % CI 1·23, 3·12), 4 months (95 % CI 1·28, 5·77) and 6 months of age (95 % CI 1·54, 9·75) compared to the infants of the food-secure women, while they were also 1·85 times more likely to be underweight at the age of 4 months (95 % CI 1·01, 3·41). After adjustment for pre-pregnancy BMI, age, education level, parity, smoking habits and place of living, the risk of stunting remained high at birth (adjusted relative risk (aRR) = 2·01; 95 % CI 1·17, 3·46), 4 months (aRR = 3·03; 95 % CI 1·21, 7·61), and 6 months of age (aRR = 3·83; 95 % CI 1·37, 10·68) in the infants born in food-insecure households compared to those in food-secure households. On the other hand, food insecurity was not significantly associated with weight-for-height Z score and WAZ during the first 6 months of life, and no correlation was observed with birth weight. These results remained persistent after adjustment for potential confounders.

**Table 4:** Unadjusted and adjusted relative risk of household food insecurity during pregnancy in anthropometric characteristics of neonates (number of percentage (%) and RR and 95 % CI values)

				Unadjusted			Adjusted		
		Food security (%)		RR (95 % CI			RR (95 % CI)		
Characteristics		No (%)	Yes (%)	RR	95 % CI	***P*****-value**	RR	95 % CI	***P*****-value**
Birth weight	LBW	4·6	3·7	1·26	0·83, 1·90	0·274	1·10	0·67, 1·83	0·706
	Normal	90·5	92	Referent					
	Macrosomia	4·9	4·3	1·16	0·78, 1·72	0·470	1·22	0·78, 1·90	0·378
Birth WHZ	Wasted	8·3	8·4	0·96	0·68, 1·35	0·180	0·94	0·64, 1·37	0·746
	Normal	90	90·2	Referent					
	Overweight	2·4	1·4	1·38	0·67, 2·83	0·382	1·02	0·47, 2·20	0·958
2-month WHZ	Wasted	4	4·2	0·95	0·61, 1·47	0·821	0·95	0·59, 1·53	0·839
	Normal	92	92·6	Referent					
	Overweight	4·0	3·2	1·25	0·79, 1·97	0·337	1·09	0·66, 1·79	0·750
4-month WHZ	Wasted	2·1	3·5	0·59	0·33, 1·03	0·134	0·64	0·34, 1·20	0·159
	Normal	94·7	92·9	Referent					
	Overweight	3·3	3·6	0·90	0·57, 1·46	0·667	0·82	0·50, 1·36	0·439
6-month WHZ	Wasted	2·5	2·5	0·99	0·53, 1·84	0·667	1·25	0·64, 2·43	0·511
	Normal	94·1	93·3	Referent					
	Overweight	3·5	4·2	0·81	0·48, 1·35	0·413	0·88	0·50, 1·53	0·640
Birth WAZ	Underweight	3·4	2·3	1·47	0·84, 2·58	0·180	1·71	0·84, 3·48	0·136
	Normal	94·6	96·3	Referent					
	Other	2	1·4	1·48	0·72, 3·07	0·291	1·07	0·49, 2·35	0·862
2-month WAZ	Underweight	3·5	2·6	1·34	0·82, 2·19	0·248	1·34	0·80, 2·45	0·244
	Normal	92·9	94·7	Referent					
	Other	3·6	2·7	1·35	0·83, 2·20	0·220	1·25	0·74, 2·12	0·412
4-month WAZ	Underweight	2·6	1·4	1·85	1·01, 3·41	0·049	1·81	0·91, 3·61	0·094
	Normal	94·3	95·1	Referent					
	Other	3·1	3·4	0·92	0·56, 1·51	0·743	0·85	0·51, 1·42	0·539
6-month WAZ	Underweight	2·3	1·6	1·58	0·76, 3·27	0·271	1·56	0·71, 3·44	0·272
	Normal	94·4	94·4	Referent					
	Other	3·5	4·3	0·82	0·49, 1·37	0·443	0·89	0·51, 1·55	0·666
Birth HAZ	Stunted	5·7	3	1·96	1·23, 3·12	**0·005**	2·01	1·17, 3·46	**0·012**
	Normal	94·2	96·7	Referent					
	Other	0·2	0·3	0·49	0·06, 4·39	0·524	0·51	0·05, 4·98	0·559
2-month HAZ	Stunted	3·4	2·9	4·46	0·98, 21·53	0·053	1·14	0·66, 1·96	0·632
	Normal	96·3	96·1	Referent					
	Other	0·3	1	3·95	0·91, 17·20	0·068	0·27	0·06, 1·23	0·092
4-month HAZ	Stunted	2·1	0·8	2·72	1·28, 5·77	**0·009**	3·03	1·21, 7·61	**0·018**
	Normal	97·4	98·5	Referent					
	Other	0·5	0·7	0·74	0·24, 2·33	0·608	0·56	0·15, 2·10	0·386
6-month HAZ	Stunted	2·1	0·6	3·87	1·54, 9·75	**0·004**	3·83	1·37, 10·68	**0·010**
	Normal	97·2	98·5	Referent					
	Other	0·7	1	0·69	0·22, 2·16	0·529	0·85	0·25, 2·86	0·795

Multinomial logistic regression adjusted for maternal age, pre-pregnancy BMI, education level and parity; *P* < 0.05 considered statistically significant. HAZ, height-for-age Z scores; LBW, low birth weight; WAZ, weight-for-age Z scores; WHZ, weight-for-height Z scores.

## Discussion

Despite the fact that HFI has been associated with several adverse health outcomes among children, there is scarce literature regarding the correlation of HFI during pregnancy with the nutritional status of children. This was the first study to determine the effects of HFI during pregnancy on infants’ growth indicators in Iran. Our findings indicated that the mean WAZ and HAZ were significantly lower in infants aged 6 months born to food-insecure mothers. HFI during pregnancy may be a risk factor for stunting in infants aged less than 6 months. However, no significant association was observed between maternal food insecurity and birth weight in this study.

Our results showed that the children of food-insecure households had lower WAZ and HAZ compared to the food-secure ones, which is consistent with the studies conducted in other low- and middle-income countries, such as South Ethiopia^(33)^, Nepal^(34)^, Brazil^(16)^ and Kenya^(15)^. Another study conducted on children of different age groups in the same region reported that HFI was significantly correlated with the mean height of the infants aged less than 6 years^(35)^. It seems that food insecurity could affect child development in the early stages of life through at least two mechanisms. First, food-insecure mothers have limited access to nutritionally adequate and safe food, which decreases the resources for the proper growth of their infants during pregnancy and breastfeeding^(36,37)^. Another mechanism is via the maternal emotional status, as food insecurity could be the driving force behind the disruption of maternal psychological and hormonal balance, which in turn has an adverse impact on the quality and duration of breastfeeding^(38,39)^.

The current research’s initial finding was that HFI was only associated with child stunting, and no correlations were observed with wasting and underweight. Stunting or chronic malnutrition is often an indication of long-term nutritional deprivation. However, this is an issue of greater magnitude than underweight or wasting, more accurately reflecting the nutritional deficiencies and illnesses that occur in the most critical periods of growth and development in the early stages of life^(28)^. This impaired linear growth, during the first 1000 d of life particularly, tends to remain up to adulthood, which is associated with greater risk of morbidity and mortality^(40)^, poor cognition and educational performance^(41)^ and nutrition-related chronic diseases in adult life^(42)^.

A growing body of evidence shows that HFI is closely linked to greater risk of stunting in early age in developing countries. A study conducted in South Ethiopia reported that children aged 6–59 months living in food-insecure households were 6·7 times more at risk of stunting than their counterparts in food-secure households^(33)^. Likewise, A cross-sectional study on 2591 children aged 0–60 months confirmed the association of HFI with increased risk of stunting even after adjusting for child, mother and household confounders^(43)^. In a multi-country study conducted on 800 households in eight countries, Psaki et al. found that food access insecurity was associated with a significant shift in the distribution of children’s HAZ toward lower values; the risk of stunting increased by 12 % among children from food-insecure households.

Since the growth of infants aged less than 6 months largely depends on the nutritional status of mothers through breastfeeding, the adverse conditions in this period may even deteriorate with the initiation of complementary feeding^(44)^. Hence, our findings highlight this point that there is a clear need to establish early and timely preventive actions to address HFI during pregnancy and the first 6 months after birth.

In contrast to our finding regarding the lack of an association between food insecurity during pregnancy and birth weight, the study by Saeed et al. on 103 pregnant women aged 19–45 years indicated that food-insecure women were at a fivefold increased risk of delivering low birth weight neonates^(45)^. Moreover, a prospective cohort study of 5044 pregnant women showed that severe HFI is a risk factor for the higher rate of low birth weight^(46)^. The discrepancy could be because the method used in the mentioned study to assess the HFI status differed from our research. In addition, the rate of food insecurity in our population was low at baseline.

There are some limitations that should be acknowledged. Firstly, there was a lack of access to reliable data on the households’ income status and the inevitable use of the habitation area as a welfare indicator. Secondly, our study was only focussed on the first 6 months of life to clarify the linkage between food insecurity during pregnancy and the growth indicators of infants when they primarily depended on the maternal nutritional status. Therefore, further longitudinal studies are recommended on various age groups in order to confirm the associations. Notably, this was the first multi-centric study regarding the effects of HFI on the growth indicators of infants aged less than 6 months in Iran.

## Conclusion

The results of this longitudinal study indicated that food insecurity during pregnancy might be a risk factor for stunting in infants aged less than 6 months. Our findings could be incorporated into nutritional interventions during and before pregnancy as HFI may have detrimental effects on children’s growth and development in the early stages of life, which could become life-long.
